# Evaluation of the clinical outcomes of telehealth for managing diabetes: A PRISMA-compliant meta-analysis

**DOI:** 10.1097/MD.0000000000012962

**Published:** 2018-10-26

**Authors:** Cong Wu, Zixiang Wu, Lingfei Yang, Wenjun Zhu, Meng Zhang, Qian Zhu, Xiaoying Chen, Yongmiao Pan

**Affiliations:** a Department of Medical Quality Management, The Women's Hospital; b Department of Thoracic Surgery, The Second Affiliated Hospital, Zhejiang University School of Medicine, Hangzhou, Zhejiang, China.

**Keywords:** clinical outcomes, diabetes management, meta-analysis, systematic review, telehealth

## Abstract

**Introduction:**

The objective of this study was to systematically review the literature and perform a meta-analysis comparing the clinical outcomes of telehealth and usual care in the management of diabetes.

**Methods:**

Multiple strategies, including database searches (MEDLINE, PsycINFO, PubMed, EMBASE, and CINAHL), searches of related journals and reference tracking, were employed to widely search publications from January 2005 to December 2017. The change in hemoglobin A1c (HbA1c) levels was assessed as the primary outcome, and changes in blood pressure, blood lipids, body mass index (BMI), and quality of life were examined as secondary outcomes.

**Results:**

Nineteen randomized controlled trials (n = 6294 participants) were selected. Telehealth was more effective than usual care in controlling the glycemic index in diabetes patients (weighted mean difference = −0.22%; 95% confidence intervals, −0.28 to −0.15; *P* < .001). This intervention showed promise in reducing systolic blood pressure levels (*P* < .001) and diastolic blood pressure levels (*P* < .001), while no benefits were observed in the control of BMI (*P* = .79). For total cholesterol and quality of life, telehealth was similar or superior to usual care.

**Conclusion:**

Telehealth holds promise for improving the clinical effectiveness of diabetes management. Targeting patients with higher HbA1c (≥9%) levels and delivering more frequent intervention (at least 6 times 1 year) may achieve greater improvement.

## 1 Introduction

The World Health Organization (2016) stated that 422 million adults were estimated to suffer from diabetes worldwide in 2014, and the prevalence of this disease has almost doubled since 1980, growing from 4.7% to 8.5%. In 2012, 1.5 million deaths were directly caused by diabetes globally, and an additional 2.2 million patients who died of cardiovascular and other diseases were associated with higher than optimal blood glucose levels.^[1]^ Due to the direct medical costs of diabetes and being unemployed, this disease leads to substantial economic losses for diabetes patients and their families, for health organizations, and for nations.^[1]^ Thus, with a high incidence, high mortality, long-term impacts on health and large diabetes-related expenditures, this disease has become a great concern for human beings.

Romanow^[2]^ demonstrated that well-designed management of diabetes and modifiable risk factors can potentially prevent disease progression, complications, and premature death from diabetes. However, because of limited health resources, the absence of self-management education and insufficient recommendations from professionals concerning medication adjustments and lifestyle changes in many remote areas; it is difficult for diabetes patients to achieve effective management. Telehealth is an innovation that allows medical professionals to diagnose patients in a distant area through the exchange of monitoring results and the delivery of healthcare services via electronic communication (fax, Internet, modem, telephone, or mobile phone).^[3–6]^ This innovation aims to overcome the barriers of health service access for people in rural areas and to assist patients in better understanding their health conditions, encourage self-management of health problems, and alert professionals to provide support when needed.^[7]^

Despite the obvious promise of telehealth, the clinical and cost effectiveness of this innovation remain poorly documented.^[8]^ In the few reported studies, the results of telehealth were variable; some studies showed that telehealth could generate statistically significant improvements in clinical outcomes, diabetes-related expenditures, hospital admissions and hospitalizations,^[9–11]^ and other studies have claimed that telehealth is similar to usual care for diabetes control.^[12,13]^ Thus, the objective of this systematic review was to compare the clinical outcomes of telehealth and usual care in the management of diabetes. This study focused mainly on trials with a large sample size (n > 100) and a long-term intervention (duration > 6 months) to provide rigorous evidence for policy makers.

## 2 Methods

### 2.1 Literature search strategy

Multiple strategies, including database searches, searches of related journals, and reference tracking, were employed to widely search publications from January 2005 to December 2017. This study was reviewed and approved by the Ethics Committee of the Women's Hospital of Zhejiang University. The electronic databases of MEDLINE, PsycINFO, PubMed, EMBASE, and CINAHL were initially searched for combinations of the following Key words: tele∗/e-health/m-health/web-based/internet-based/online/phone/mobile application/remote care/computer, diabetes/diabetes mellitus and treatment outcom∗/clinical eff∗/clinical outcom∗/treatment effect∗. Then, the reference lists of the relevant literatures and key journals were manually searched to identify additional publications.

### 2.2 Criteria for study inclusion and exclusion

Randomized controlled trials (RCTs) that examined the clinical outcomes of telehealth interventions in adults with type 1 or type 2 diabetes compared with those of conventional care were included. The telehealth intervention was required to include one or more of the following categories: tele-education, telemonitoring, teleconsultation, telecase management, and telementoring.^[3]^ Trials that were reported in non-English publications, involved <100 participants in the initial recruitment, reported <6 months of follow-up, or investigated gestational diabetes were excluded. Research protocols and substudies of the included studies were also excluded.

### 2.3 Methods of study selection

Duplications automatically detected by document manager (NoteExpress) were removed first. The titles and abstracts that were obtained through a literature search were scanned independently by 2 reviewers, and potential studies were identified through comparison with the selection criteria. Then, the full texts of these articles were reviewed, and those that were not consistent with the objectives were excluded. The results of 2 reviewers were compared, and any differences were discussed and resolved by consensus.

### 2.4 Data extraction and quality assessment

Data from the selected studies were independently extracted by 2 reviewers by using a data extraction form. A risk-of-bias assessment tool, summarized in the Cochrane Handbook for Systematic Reviews of Interventions (version 5.1.0), was applied to assess the quality of each study.^[14]^ The studies were evaluated separately based on 7 domains: random sequence generation; allocation concealment; blinding of participants and personnel; blinding of outcome assessment; incomplete outcome data; selective reporting; and other bias. Two reviewers subjectively reviewed all the selected studies and assigned a value of “Low risk,” “Unclear risk,” or “High risk” to these 7 domains in the Review Manager software (version 5.3).

### 2.5 Outcome measures

The primary outcome was the change hemoglobin A1c (HbA1c), pre- and postintervention because this parameter has been regarded as a gold standard indicator of clinical outcomes in diabetes. This parameter reflects the mean glycemia level in the past 2 to 3 months and is strongly associated with complications of diabetes.^[15]^ Changes in blood pressure, blood lipids, body mass index (BMI), and quality of life, which are important clinical outcomes representing the ultimate goal of treatment,^[16]^ were the secondary outcomes in this study.

### 2.6 Data analysis methods

The statistical analyses in this systematic review were all performed in Review Manager software (version 5.3). Differences in means, with 95% confidence intervals (CIs), were measured to compare the telehealth group with the usual care group to determine the changes in HbA1c, blood pressure, blood lipids, BMI, and quality of life. All data expressed in terms of the median and range were converted to means and standard deviations by applying the Hozo approach.^[17]^ Study heterogeneity was measured by the I^2^ statistic, which presented the percentage of the total variability among the studies that was caused by heterogeneity rather than chance.^[18]^ When heterogeneity was substantial (I^2^ > 50%), a sensitivity analysis was performed to identify studies with significant differences; only if the sensitivity analysis was ineffective, a random-effects model was applied rather than a fixed-effects model. *P* values were calculated by comparing the resulting statistic with a chi-squared distribution, and statistically significant differences were identified when *P* values < .05.

## 3 Results

### 3.1 Study characteristics

**Figure 1:**
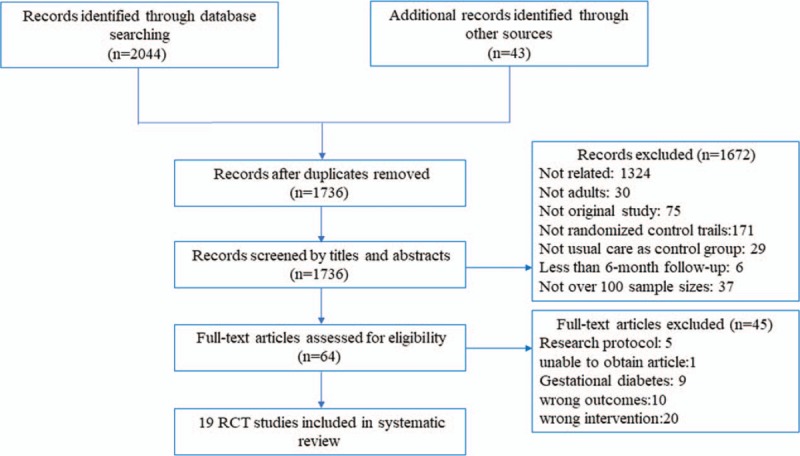
The flow of study selection.

As shown in the flowchart (Fig. 1), 19 publications were finally selected for inclusion. The characteristics of these 19 studies were summarized in Table 1. Most of them were single-center studies that occurred in the United States^[8,19–27]^ or Europe.^[28–34]^ The sample size ranged from 100^[22,28]^ to 1665 participants.^[8]^ Among the 6294 participants, 3269 were randomized to the telehealth group, while 3025 were chosen as members of the usual care group. The length of the intervention varied from 6 to 12 months. All the studies were conducted in adults: the mean age ranged from 45.5 to 68.4 years in the telehealth group and 50.9 to 67.9 years in the usual care group. The participants selected in most studies were those who were able to self-monitor blood glucose and could use technology (e.g., computer or telephone) to interact with their healthcare providers. Volunteers with cognitive dysfunction; reading and listening barriers; severe life-threatening illness were excluded.

**Table 1:**
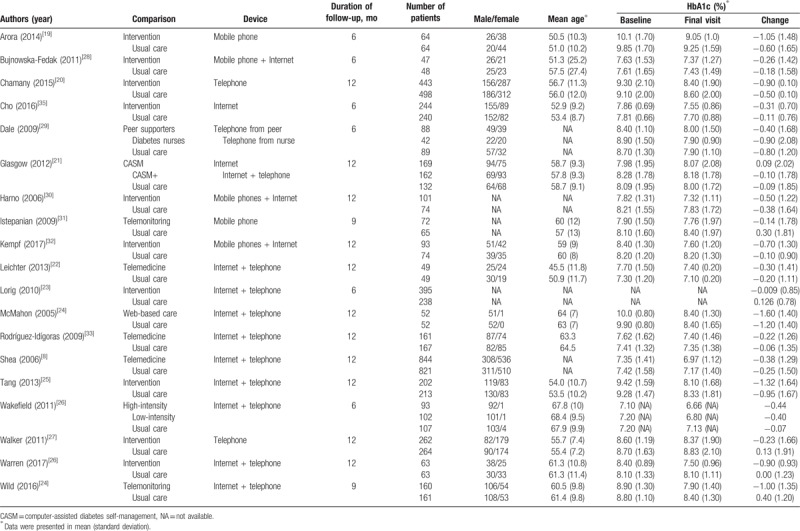
Summary of the studies and glycated hemoglobin in each study.

The telehealth intervention in most of the selected trials involved self-monitoring of blood glucose and data transmission, either manually or electronically, with feedback (n = 14)^[8,22–26,28,30–36]^; in the remaining studies, the procedures were not specifically mentioned. Mobile phone, telephone, Internet, modem, or Bluetooth communication was employed to transmit monitoring data in these 14 studies. Most studies required the participants to monitor and transmit the data weekly or less than a week and provided feedback through text messages, standardized messages, phone calls, Internet-based communications, a website or email. Generally, the feedback included advice on medication adjustments, a healthy diet and physical activity. The approaches used to deliver education included telephone calls,^[19,20,22,27,29,32]^ web-based educational modules,^[21,23,24,26,31,35]^ Internet-based communication,^[25,35]^ videoconferencing,^[8,36]^ and short message service.^[30]^ Education was mostly administered by a multidisciplinary team that consisted of nurses, physicians, clinical health psychologists, diabetologists, or exercise experts (n = 7),^[8,20,24,26,28,29,35]^ while others were simply by nurses,^[27,34,36]^ clinicians,^[8,31]^ or endocrinologists.^[22]^ All of these educational strategies aimed to enhance patient motivation, self-efficacy, and self-management ability.

### 3.2 Quality assessment

**Figure 2:**
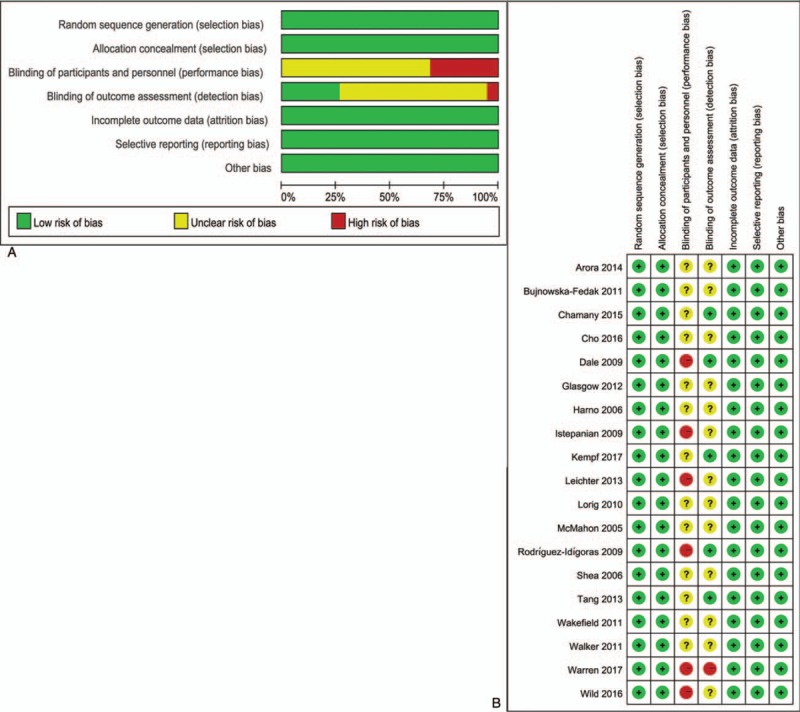
(A) A risk-of-bias assessment of all included studies; (B) a risk-of-bias assessment of individual included study.

Figure 2A summarizes the quality of entire included trials in this study, while Fig. 2B presents the quality of the individual trials included. As shown in Fig. 2A, the allocation sequence was randomly generated in all trials. Every study had reported the concealment of the allocation and addressed incomplete outcome data adequately. Due to the nature of telehealth, it was impossible for the patients to be blinded to their allocation. However, some trails were designed such that the patient allocation remained unknown to the outcome assessors.

### 3.3 Diabetic control outcomes

#### 3.3.1 HbA1c

**Figure 3:**
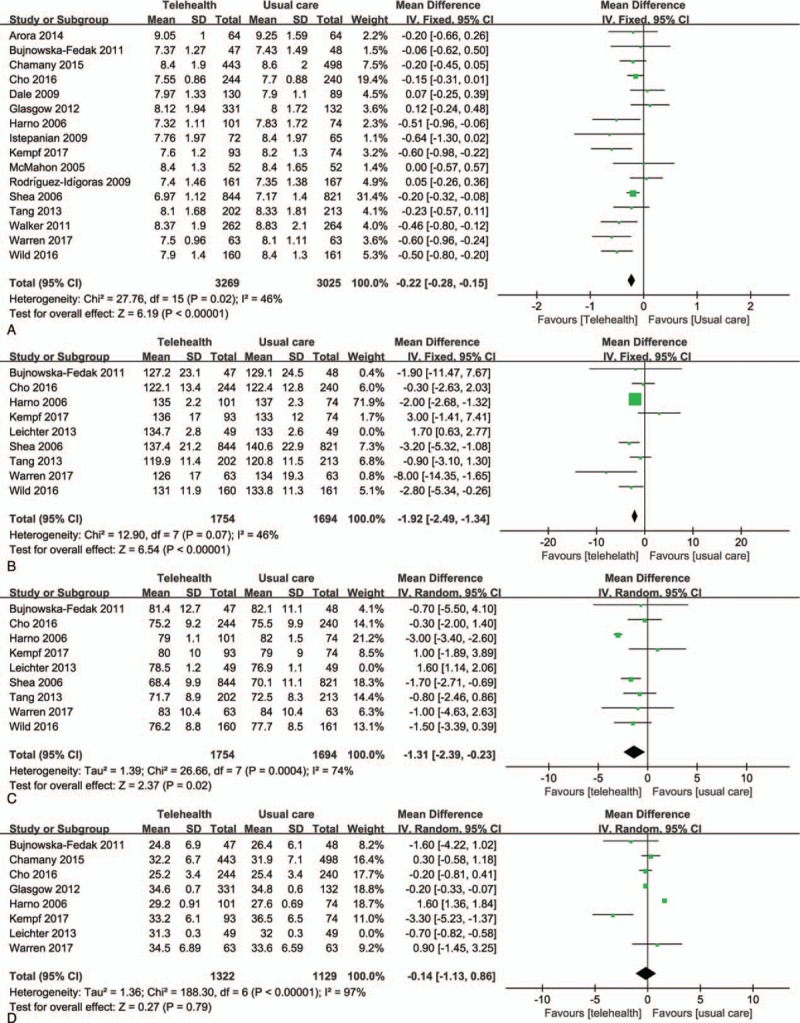
Effect of telehealth on clinical outcomes of diabetes. (A) Forest plot showing the results of the meta-analysis of glycemic control; (B) forest plot showing the results of the meta-analysis of systolic blood pressure change; (C) forest plot showing the results of the meta-analysis of diastolic blood pressure change; (D) forest plot showing the results of the meta-analysis of body mass index change. CI = confidence interval.

The data from 16 RCTs were pooled to find the effects of diverse telehealth approaches on HbA1c. As shown in Fig. 3A, the HbA1c levels in the telehealth group were significantly lower than those in the usual care group (weighted mean difference = −0.22%; 95% CI, −0.28 to −0.15; *P* < .001). The statistical heterogeneity was low (I^2^ = 46%), and a fixed-effects model was used in this analysis. As in the studies by Lorig et al^[23]^ and Wakefield et al,^[26]^ data for the mean or standard deviation at the final visit were not given, these 2 studies were not pooled with the other studies. Both studies found that HbA1c was improved in the telehealth group compared with the usual care group, while no significant between-group differences were observed in the study by Leichter et al,^[22]^ which was not pooled with other studies because of heterogeneity. In addition, Table 1 shows that the average change in HbA1c in the telehealth group was approximately −1.22% when the baseline level of the participants was 9.0% or above, and the average change in HbA1c was approximately −0.35% when the baseline level was lower than 9.0%.

#### 3.3.2 Blood pressure

Blood pressure was reported in 9 studies, and the results are presented in Table 2. Eight of these studies were pooled and examined both systolic blood pressure (Fig. 3B) and diastolic blood pressure (Fig. 3C). Figure 3B and C shows a statistically significant decrease in systolic blood pressure (weighted mean difference = −1.92; 95% CI, −2.49 to −1.34; *P* < .001) and diastolic blood pressure (weighted mean difference = −1.31; 95% CI, −2.39 to −0.23; *P* < .001) in the telehealth group compared to the usual care group. As the statistical heterogeneity was higher than 50%, a random-effects model was applied in the meta-analysis of diastolic blood pressure.

**Table 2:**
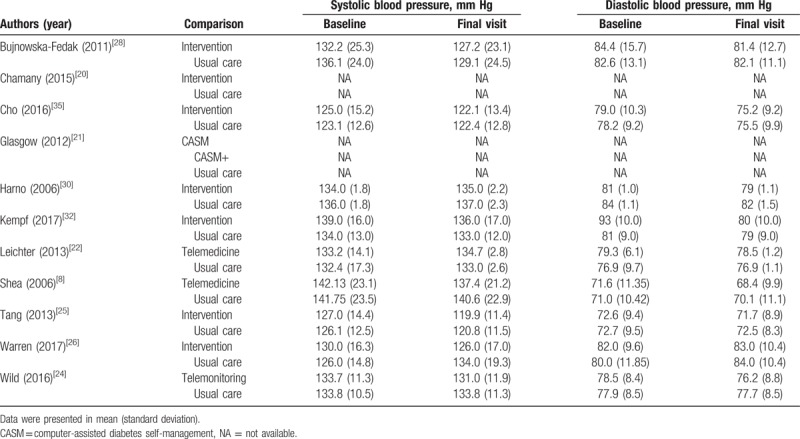
Summary of the systolic blood pressure and diastolic blood pressure in studies.

#### 3.3.3 BMI, total cholesterol, and quality of life

BMI was reported in 10 studies (Table 3) and Fig. 3D shows that there was no significant difference between the telehealth group and the usual care group in controlling BMI (weighted mean difference = −0.14; 95% CI, −1.13 to 0.68; *P* = .79). Six included trials reported the total cholesterol outcomes, as shown in Table 3. Only 2 trials^[8,30]^ reported that the total cholesterol was significantly lower in the telehealth group than that in the usual care group; 4 studies^[28,32,35,36]^ showed a nonsignificant difference between this 2 groups over the duration of follow-up. The outcomes of quality of life were not pooled because the measurement instruments used in these trials varied significantly. Two studies^[21,28]^ stated that quality of life improved in the telehealth group while no statistically significant difference was found; 1 study^[32]^ showed that the impairment of quality of life decreased (*P* < .001) in the telehealth group versus the usual care group.

**Table 3:**
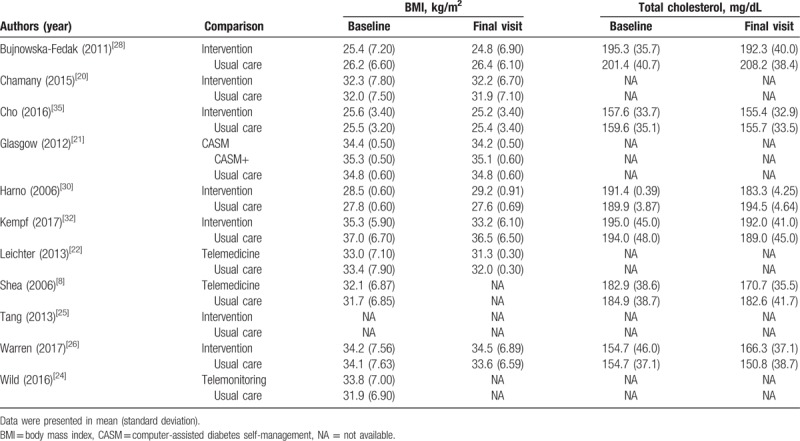
Summary of the body mass index and total cholesterol in studies.

## 4 Discussion

The results of this meta-analysis indicated that compared with usual care, telehealth had a positive effect on glycemic and blood pressure control, while no significant difference was found in the control of BMI. For total cholesterol and quality of life, telehealth was similar or superior to usual care.

The results of this review related to HbA1c control are generally consistent with some previous reviews. Liang et al^[37]^ demonstrated that telehealth intervention reduced HbA1c by a mean of 0.5% (*P* < .001); Zhai et al^[11]^ found a lower HbA1c level in the telehealth group (*P* < .001) than that in the usual care group; and Lee et al^[3]^ showed that telehealth improved HbA1c by −0.18% (*P* = .01). DelliFraine and Dansky^[38]^ failed to support a link between telehealth and diabetes outcomes (the effect size was 0.13; Z = 1.3). In this study, the sample size ranged from 31 to 141, and the evidence base for HbA1c was limited (only 5 included studies studied HbA1c). Moreover, participants with other serious diseases were not excluded in the original studies, which could potentially affect the accuracy of the results. Verhoeven et al^[13]^ also found no significant difference between the teleconsultation and usual care groups after conducting a meta-analysis of 6 RCTs (*P* = .82). In this meta-analysis, the duration of follow-up was 3 to 4 months in the original trails, which was not long enough to capture valid data. As Lee et al^[3]^ illustrated, a longer duration (>6 months) could result in larger effects. In our meta-analysis, the sample size ranged from 100 to 1665, and the duration of follow-up was at least 6 months, which may provide more effective evidence.

Participants with higher baseline HbA1c levels (≥9%) may be associated with greater effects when receiving a telehealth intervention. The results of this study showed that the average HbA1c change in the recruited patients with higher baseline HbA1c levels (≥9%) was larger than that in patients with lower baseline HbA1c levels (<9.0%) (−1.22% vs −0.35%). This difference may have occurred because participants with higher baseline levels had poorer self-care management, relating to healthy eating, exercise adherence as well as medication administration.^[39]^ Telehealth offered a mechanism to improve medication administration, such as regular reminders and adjustments of the medication dose for patients when needed. Benefiting from information and communication technologies, telehealth introduced high-quality diabetes self-management education to individuals who lived in remote areas, which can facilitate patients’ healthy eating, exercise adherence and self-monitoring of blood glucose. Thus, telehealth could potentially enhance self-care management for participants and result in lower HbA1c levels. Lee et al^[3]^ also illustrated, higher baseline HbA1c (≥9%) levels were related to larger effects after the telehealth intervention. Therefore, it can be speculated that targeting patients with higher HbA1c (≥9%) levels with telehealth interventions could achieve greater effects. Different intervention frequencies may influence the effect of telehealth. In the study conducted by Leichter et al,^[22]^ the endocrinologist analyzed the transmitted data and provided feedback regarding treatment changes via the Internet and telephone only during the 3rd and 9th months of the study period. The remaining intervention was the same as usual care. Eventually, no superior effect was found in the telehealth group over the usual care group in this study. However, in most of rest studies, the intervention frequency ranged from weekly to monthly. For instance, in the study of Wild et al^[34]^ participants received suggestions on lifestyle modifications and treatment adjustments weekly from the primary care nurses based on the participants’ results. This study found that telehealth intervention reduced HbA1c by a mean of 0.51% (*P* < .001) compared with that in the usual care group. Additionally, Walker et al^[27]^ demonstrated that 6 times interventions in 12 months were the minimum frequency associated with a significant decrease in HbA1c. Therefore, we speculated that frequent intervention (at least 6 times 1 year) may result in better outcomes, or the usefulness of telehealth could be weakened. Further prospective and randomized studies are needed to identify the telehealth strategies and protocols that would be most beneficial for patients.

The results of this study demonstrated that blood pressure significantly decreased in the telehealth group (*P* < .001), while no significant difference in BMI (*P* = .79) was found. Among the pooled studies, most of the mean baseline systolic blood pressures were above 130 mm Hg but under 140 mm Hg, which cannot be diagnosed with hypertension. Because all the included studies mainly targeted the participants with diabetes, whose HbA1c levels were abnormal, their blood pressure, BMI, and blood lipid levels were not sure to be under control. Thus, the difference in effects on systolic blood pressure between the telehealth and usual care groups was probably larger when targeting the participants with hypertension. The results obtained for diastolic blood pressure and BMI should be interpreted with caution due to the high level of heterogeneity (diastolic blood pressure, I^2^ = 74%; BMI, I^2^ = 97%). Other outcomes, including total cholesterol and quality of life, were limited and reported in only 6 and 3 studies, respectively. More studies should measure these important outcomes to draw an explicit conclusion regarding the utilization of telehealth interventions.

The findings of this study held promise in supporting telehealth practice and policy. Stratton et al^[40]^ demonstrated that for type 2 diabetes patients, reducing the 1% mean HbA1c level would be related to a 21% reduction in diabetes-related death and a 37% reduction in microvascular complications, such as neuropathy, retinopathy, and blindness. As mentioned above, compared with usual care, telehealth could achieve a 0.22% mean HbA1c reduction, and it could be speculated that approximately 170,940 diabetes-related deaths could have been avoided, if this intervention was implemented in 2012; because a total of 3.7 million deaths were associated with blood glucose levels.^[1]^ Therefore, it is worth promoting the adoption and sustainability of this innovation by policy makers.

There are several limitations to this systematic review. First, only 25% of the included studies showed successful blinding of the outcome assessment, which may lead to performance and detection bias. Second, only limited guidance about the outcomes of telehealth in managing diabetes could be provided by this meta-analysis. Several areas need further clarification. For instance, it would be helpful to identify whether the effects of telehealth are influenced by the frequency and pattern of data delivery, the strength and mode of intervention, the baseline level of the indicator and the target participants. Further research should be conducted to provide more valid evidence for the effects and sustainable implementation of telehealth.

## 5 Conclusion

The findings showed evidence that telehealth holds promise in improving the clinical effectiveness of diabetes management. Targeting patients with higher HbA1c (≥9%) levels and delivering more frequent intervention (at least 6 times 1 year) may achieve greater improvement.

## Author contributions

**Conceptualization:** Cong Wu, Zixiang Wu, Lingfei Yang, Meng Zhang, Xiaoying Chen, Yongmiao Pan.

**Data curation:** Cong Wu, Zixiang Wu, Lingfei Yang, Xiaoying Chen, Yongmiao Pan.

**Formal analysis:** Cong Wu, Zixiang Wu, Qian Zhu.

**Investigation:** Wenjun Zhu.

**Methodology:** Cong Wu, Zixiang Wu, Yongmiao Pan.

**Project administration:** Yongmiao Pan.

**Resources:** Cong Wu.

**Supervision:** Cong Wu, Meng Zhang, Yongmiao Pan.

**Writing – original draft:** Cong Wu, Zixiang Wu, Lingfei Yang, Wenjun Zhu, Meng Zhang, Qian Zhu, Xiaoying Chen, Yongmiao Pan.

**Writing – review & editing:** Cong Wu, Zixiang Wu, Lingfei Yang, Wenjun Zhu, Meng Zhang, Qian Zhu, Xiaoying Chen, Yongmiao Pan.
